# Effect of Thermal Aging on the Physico-Chemical and Optical Properties of Poly(ester urethane) Elastomers Designed for Passive Damping (Pads) of the Railway

**DOI:** 10.3390/polym13020192

**Published:** 2021-01-07

**Authors:** Liliana Rosu, Cristian-Dragos Varganici, Dan Rosu, Stefan Oprea

**Affiliations:** 1 Centre of Advanced Research in Bionanoconjugates and Biopolymers, “Petru Poni” Institute of Macromolecular Chemistry, 41A Gr. Ghica-Voda Alley, 700487 Iasi, Romania; lrosu@icmpp.ro; 2 Laboratory of Polyaddition and Photochemistry, “Petru Poni” Institute of Macromolecular Chemistry, 41A Gr. Ghica-Voda Alley, 700487 Iasi, Romania; stefop@icmpp.ro

**Keywords:** poly(ester urethane)s, FTIR, thermal aging, physico-chemical properties, lifetime prediction

## Abstract

The aim of this study consists of monitoring the effect of thermal aging on the physico-chemical and optical properties of poly(ester urethane) elastomers designed as damping materials for railways. The materials were obtained by polyaddition in two stages in melt, resulting in regular structures. The structural modifications during the thermal aging of the samples were monitored using FTIR, color changes, TGA in non-isothermal and isothermal conditions, DSC and physico-mechanical measurements. The structural regularity of the rigid and flexible segments maintained the good mechanical properties of the structures up to 200 h of thermal aging at the elevated temperatures of 40 °C, 70 °C, 100 °C and 130 °C. It was observed that at 40 °C and low exposure times, changes occur mainly to the carbonyl groups of the soft segments. At higher temperatures and longer exposure times urethane groups were affected. Extended thermal aging led to significant changes in thermo-mechanical and optical properties.

## 1. Introduction

Polyurethanes (PURs) are a class of polymers containing the urethane segments (–NHCOO–) in the main chains. This structure assures very good chemical resistance and mechanical properties [1,2,3,4,5,6]. Polyurethane elastomers (PUREs) have known increasing interest because of their efficient cost-effective production process and recyclability, as compared to vulcanized elastomers. PUREs possess elastomer characteristic properties in normal conditions and plasticize during heating, thus they may be processed by conventional methods used for thermoplastics, such as extrusion, injection, molding or calendering [7].

PURs are constituted of linear semi-crystalline multi-block high molecular weight copolymers. The copolymers are of a biphasic structure of alternating long and amorphous flexible macromolecules (soft segments) with short and rigid crystalline chains (hard segments). PURs are the result of the polyaddition reaction between: (i) polymeric linear aliphatic diols (e.g., polyester, polyether, polycarbonate diols); (ii) aliphatic diisocyanates (the most common: 1,6-diisocyanatohexane and 1,10-methylenebis(4-isocyanatocyclohexane) (HMDI)) or aromatic compounds (the most common: 1,10-methylenebis(4-isocyanatobenzene)) and (iii) chain extenders (diols with short molecular chains), with or without a catalyst, the latter in case of a low reactivity system [8,9,10].

In a first instance, the unique properties of PURs are dictated by microphase separation, generated by the mutual immiscibility between hard and soft segments, owed to thermodynamic incompatibility. Secondly, the PURs properties are also influenced by the strong intermolecular hydrogen bonding in the hard segments, with bonding energy values ranging between 12 and 36 kJ mol^−1^ [11,12]. As a consequence of phase separation, the hard segments generate ordered micro–domains in the PUR structures, forming block copolymers connected through strong covalent bonds. The highly polar hard segments lead to strong interactions among them, either generating a high level of aggregation within the solid phase or yielding (pseudo)crystalline domains dispersed within the flexible soft segment matrix, thus behaving as nano-fillers. This aspect is common in the case of hard segments from diisocyanates and diols [13].

Nonetheless, the degree of micro-phase separation is dependent on several other factors besides polarity, such as the molecular weight of soft segments, processing conditions, etc. Soft segments may also undergo (pseudo)crystallization in the PURs [14,15]. Long chained soft segments (e.g., polyester, polyether, polycarbonate) generate negative glass transition temperature (*T*_g_) values. Such soft segments not only provide the PURs with high elasticity, but also with softness and high elongation values at break. The nano-sized filler hard segments provide PURs with enhanced mechanical properties, such as rigidity and tensile strength. Additionally, the flexibility and hardness of the PURs may be adjusted over a broad palette of either raw or processed structures, thus making them highly interesting and sought materials [16,17].

Poly(ester urethane) elastomers (PEURs) are considered amongst the most important classes of PURs because they can be processed by three different methods: extrusion, injection and moulding [18,19]. The physico-mechanical properties, chemical resistance and good processability make PEURs available in many technical applications as protective coatings, adhesives, biomedicine or damping materials [20,21].

Without a chain extender, a PEUR formed directly by the reaction between a diisocyanate and a polyol is generally a product with very low physical and mechanical properties and has no microphase separation. Thus, the introduction of a chain extender can increase the length of the rigid segment, allowing the separation of the rigid segment which results in excellent physical and mechanical characteristics, such as increased modulus and glass transition temperatures of the polymer. By modifying the ratio between the polyol and extender, PURs can be designed as hard, thermoplastic or rubber, by the simple result of the variation of the content of rigid segments in the block-copolymers [22,23,24].

Although PEURs possess special properties, their practical applicability is limited by their sensitivity to temperature [25,26,27] and light [28,29,30]. Whilst the color changes on the surface of PEUR samples occur especially under the influence of light, the increase in temperature may significantly accelerate the aging processes. Several studies on predicting the service lifetime of different materials such as composite polymeric coatings [31] or application perspectives PURs [32] and accelerated climate aging of different building materials [33] have been reported.

Boubakri et al. [34] studied the impact of aging temperature on the mechanical behavior of thermoplastic PUR. Various times and aging temperatures were selected. It was concluded that the aging process caused significant changes in mechanical properties. The thermal aging led to an enhancement of modulus and tensile stress of the PUR. However, degradation of the material was observed at higher aging times and temperature.

Long elastic soft segments and the appropriate content of hard segments are necessary for obtaining PEURs with high shape memory recovery. The condition for PEURs to possess shape recovery properties is for them to have a 1:1 molar ratio between NCO and OH groups within their structures [21]. Herein there is described the investigation of accelerated thermal aging influence on the physico-chemical, thermo-mechanical properties and color changes of some cross-linked PEURs designed for passive damping pads of the railway.

## 2. Materials and Methods

### 2.1. Materials

Poly(ethylene adipate)diol (PEA) was used as soft segment, 1,6-hexanediol (HD), 1,4-butanediol (BD) were used as chain extenders and glycerin (Gly) was used as cross-linker. The hard segments constituted of 1,6-hexamethylenediisocyanate (HDI) and 4,4′-diphenyl methane diisocyanate (MDI). BD and HD were purchased from Sigma-Aldrich and used as received. PEA, MDI and HDI were purchased from Fluka (Fluka, St. Gallen, Switzerland), the latter two being used as received. PEA (M_w_ = 2000 g mol^−1^) was dried prior to use, at 120–130 °C for 2 h under a vacuum of 2 mm Hg. Dibutyltindilaurate (DBTDL) 95%, as catalyst, was purchased from Sigma-Aldrich (Sigma-Aldrich, St. Gallen, Switzerland) and used as received.

### 2.2. Methods

#### 2.2.1. Preparation of the PEURs

**Scheme 1:**
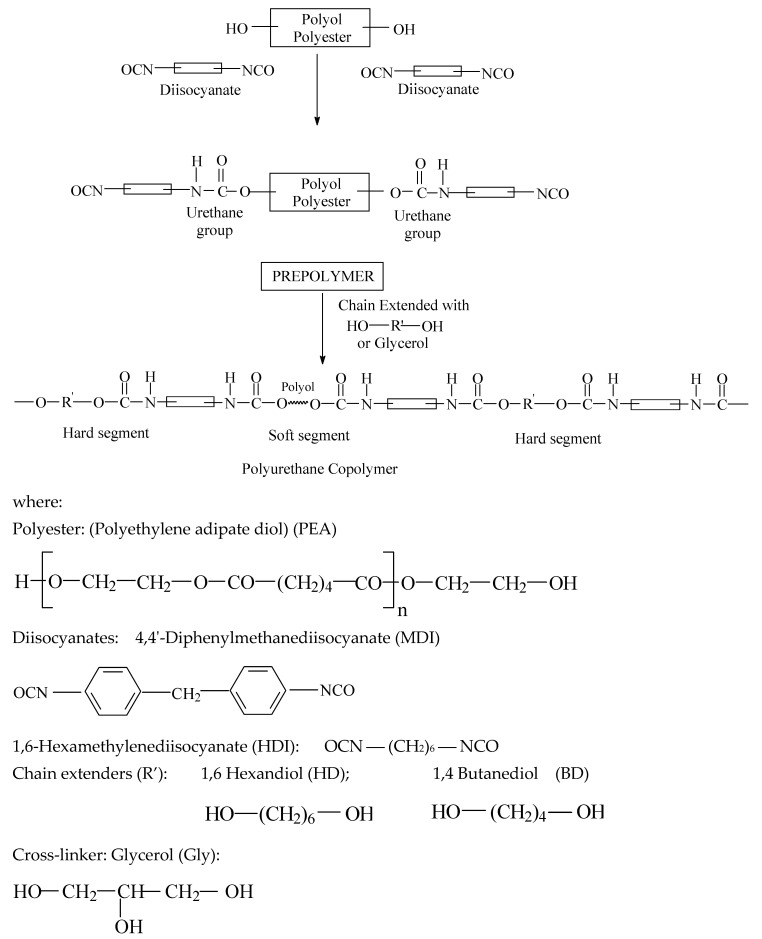
Synthesis pathways of the PEURs.

A series of six PEURs were obtained (Scheme 1). In a first stage, the precursors for the 6 PEURs were synthesized following the pre-polymer method [21]. For the synthesis of the NCO-terminated pre-polymer appropriate quantities of PEA and diisocyanate were reacted at a specified NCO/OH equivalent ratio [35]. For this purpose, these quantities were added into a 500 mL glass vessel, equipped with an oil bath, thermometer, mechanical stirrer and gas inlet and outlet through which nitrogen was purged continuously. The synthesis was conducted under stirring for 3 h at 80 °C.

The PEURs were obtained using the polyaddition technique in melt and in two steps. For this purpose, 0.01 mol of pre-polymer (polyester) was introduced into a reactor equipped with a stirrer, vacuum plant and nitrogen tube, heated up to 125 °C under stirring and a residual pressure of 2–3 mm Hg for 2 h. The temperature was lowered to 80 °C and 0.02 mol of diisocyanates (MDI or HDI) and 0.001 g of dibutyltindilaurate (DBTDL), as catalyst, were introduced under a stream of nitrogen. The reaction was continued under stirring for 1 h. Then 0.02 mol of diol or triol (BD or HD) as chain extender and Gly were introduced, with respect to MDI/PEA ratios, and stirring was continued for a short time. Films or castings were obtained from the synthesized polymer by heat treatment at 90 °C for 24 h. The reaction was monitored by FTIR (Bruker, Karlsruhe, Germany) until the band at 2200–2300 cm^−1^, corresponding to free isocyanate stretching, completely disappeared. Afterwards, the PEURs were cast into a mold and cured 24 h at 100 °C, followed by post-curing at room temperature for 7 days when they were detached in the form of sheets. The composition of the PEURs is shown in Table 1. Hard segment composition was controlled through the polyester diol/MDI/diol and triol ratios used in the synthesis, these being 1:2:1 (22% hard segment) and 1:3:2 (32% hard segment), respectively. A 1:1 ratio of OH diol/OH Gly was used during the obtaining of each PEURs. The length of the soft segment was maintained constant, while the length of hard segment was varied systematically.

#### 2.2.2. Thermal Aging

Thermal aging was performed using an oven (Memmert INB200, Borken, Germany) by heating the PEURs at different temperatures (40, 70, 100 and 130 °C) within 200 h under air atmosphere.

#### 2.2.3. Fourier–Transform Infrared Spectroscopy (FTIR)

FTIR spectra were obtained with a Bruker Vertex 70 spectrometer equipped with a Miracle^TM^ (Karlsruhe, Germany) device with a diamond crystal for attenuated total reflectance (ATR) measurements. All spectra were acquired with a spectral resolution of 4 cm^−1^.

#### 2.2.4. Color Modification Measurements

The color changes during the thermal aging of the specimens were described using the CIEL^*^a^*^b^*^ system, where L^*^ is the lightness factor, a^*^ is the redness factor and b^*^ is the yellowness factor. The L^*^, a^*^ and b^*^ parameters were measured with a color comparison device (Pocket Spec Color QA, SUA with a sensor head of 6 mm). Measurements were made using D65 illuminant and a 10° standard observer. The L^*^, a^*^ and b^*^ values were used to calculate the color change, Δ*E* as a function of the thermal aging time, according to Equation (1):

(1) $$\Delta E=\sqrt{{\left(L_{2}^{*}-L_{1}^{*}\right)}^{2}+{\left(a_{2}^{*}-a_{1}^{*}\right)}^{2}+{\left(b_{2}^{*}-b_{1}^{*}\right)}^{2}}$$

In Equation (1) indexes 1 and 2 represent the values measured before and after thermal aging. Samples were analyzed for color changes according to DIN6174 (Farbaständen bei Körperfarbennach der CIELAB–Formel, 1979).

#### 2.2.5. Thermogravimetric Analysis (TGA)

All TGA measurements (in non-isothermal and isothermal conditions) were performed on a STA 449F1 Jupiter device (Netzsch, Germany).

##### TGA in Non-Isothermal Conditions

TGA measurements in non-isothermal conditions were conducted from 30 °C up to 700 °C in nitrogen atmosphere (50 mL min^−1^) and at a heating rate of 10 °C min^−1^. Approximately 20 mg of each sample were used.

##### TGA in Isothermal Conditions

For the isothermal measurements in air, the variation of sample mass was recorded at the elevated temperatures in the aging oven. Before thermal aging, samples were dried for 24 h at 23 °C in order to remove the moisture absorption during storage.

#### 2.2.6. Differential Scanning Calorimetry (DSC)

DSC measurements were undertaken on a DSC 200 F3 Maia device (Netzsch, Germany) at heating and cooling rates of 10 °C min^−1^ and –10 °C min^−1^, in nitrogen atmosphere (50 mL min^−1^). Approximately 10 mg of each sample were used.

#### 2.2.7. Mechanical Measurements

Mechanical measurements were undertaken on dumbbell-shaped samples (ISO 37 type 2) cut from the PEURs, obtained in the form of 2 mm thick sheets. The tests were performed at 25 °C on a Shimadzu EZTest device (Tokyo, Japan), equipped with a 5 kN load cell. The cross-head speed was 50 mm min^−1^. The mechanical results were the mean of five measurements on identically shaped samples.

## 3. Results and Discussion

### 3.1. Structural Characterization by FTIR Spectroscopy

**Figure 1:**
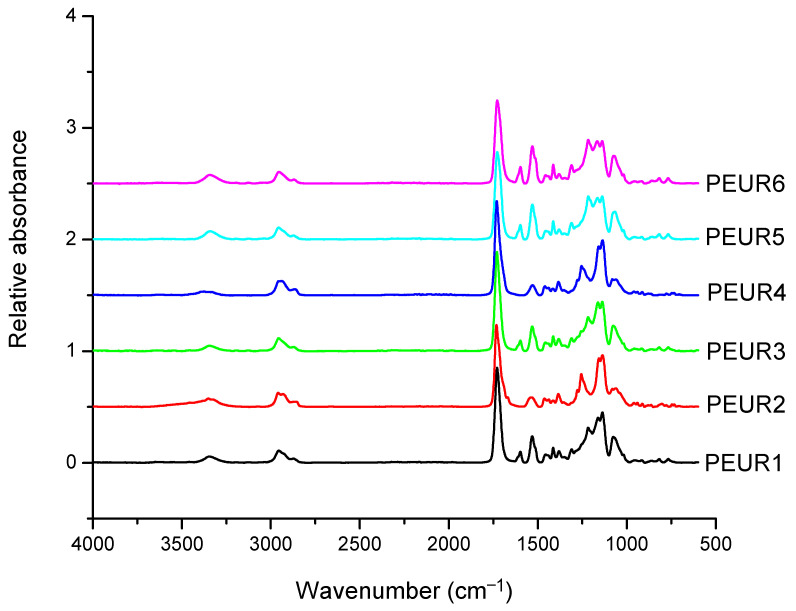
FTIR spectra of the initial PEURs.

The FTIR spectra of the obtained PEURs (Figure 1) show characteristic absorption bands: 3500–3200 cm^−1^ with a peak around 3340 cm^−1^ (free and bound –NH urethane bonds), 3000–2700 cm^−1^ (CH_2_), 1750–1650 cm^−1^ with a peak at 1730 cm^−1^ (both C=O disordered and ordered urethane bonds (1706 cm^−1^)), 1250–1050 cm^−1^ (C–O). The NH entity in PURs is characteristic to hard segments and may be conventionally used in the study of hard blocks orientation. The CH_2_ group is used for orientation of soft segments, however CH_2_ groups may also be present in low concentrations in the hard segments. Other entities include the aromatic group at 1600 cm^−1^ and –C–O–C– in the ester group at 1033 cm^−1^. The absorbance ratio 1706 cm^−1^/1730cm^−1^ increases with increasing concentration of rigid segments suggesting an increase in the proportion of bound urethane groups.

### 3.2. Properties Modifications during Thermal Aging

#### 3.2.1. Color Modifications

**Figure 2:**
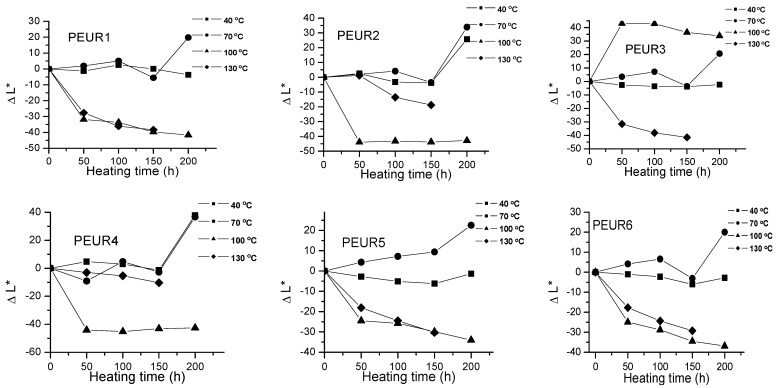
Variation of ΔL^*^ with temperature and heating time of PEURs.

**Figure 3:**
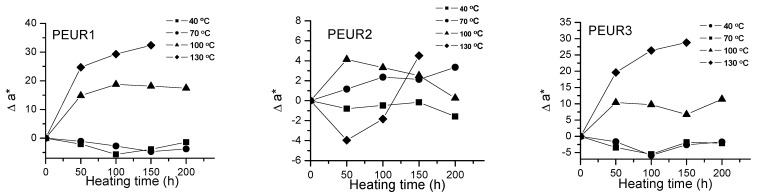

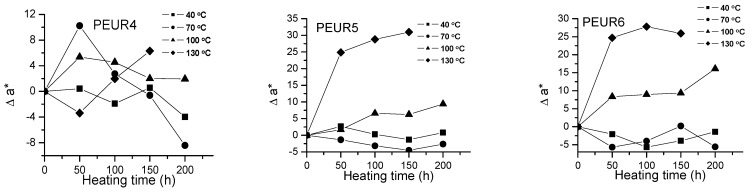
Variation of Δa^*^ with temperature and heating time of PEURs.

**Figure 4:**
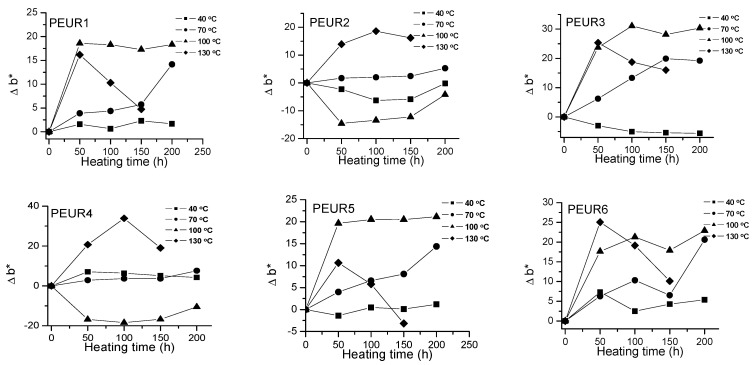
Variation of Δb^*^ with temperature and heating time of PEURs.

Important color modifications of some surface properties were observed during thermal aging. Figure 2, Figure 3 and Figure 4 and Table 2 show the color parameters variation of the PEURs as a function of temperature and heating time.

The negative ΔL^*^ values indicate surface darkening during the thermal treatment of the PEURs. For PEURs 1, 2, 3, 5 and 6 treated at 40 and 70 °C, a slight discoloring may be observed through minor increases in ΔL^*^ values. For PEUR4, up to 150 h and at 40, 70 and 100 °C, ΔL^*^ values are positive. At over 150 h of thermal aging at 100 and 130 °C the ΔL^*^ values become negative, hence the samples darken. For the thermal treatments of PEURs 1, 2, 3, 5 and 6 at 100 and 130 °C the ΔL^*^ values also become negative. For sample PEUR3 at 100 °C a strong discoloration may be observed in the first 50 h. PEUR1 darkens at temperature values below 100 °C and at lower temperatures and it lightens after 100 h. For the thermal treatment at 40 °C, ΔL^*^ values of PEUR1 vary very little with time. At 40 and 70 °C PEUR2 shows minor modifications of ΔL^*^ up to 150 h, after which it lightens, ΔL^*^ strongly increasing. For thermal treatments at 130 °C PEUR2 slowly darkens up to 150 h, after which it strongly lightens.

Red and yellow degradation intermediates accumulate on the surface of samples during the thermal treatment, as it can be seen from Figure 3, Figure 4 and Table 2. The displacements of Δa^*^and Δb^*^ to either positive or negative values do not show any pattern. This is an indication of the formation of highly instable colored intermediates during thermal aging.

**Figure 5:**
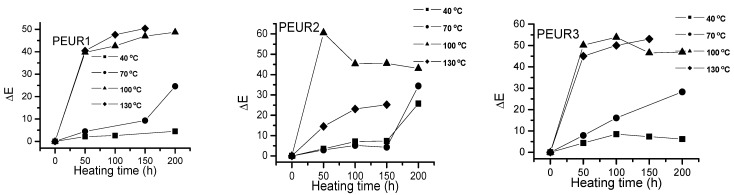

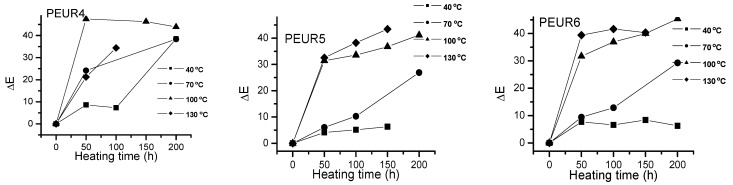
Variation of Δ*E*values with temperature and heating time for the PEURs.

Figure 5 shows the Δ*E* variation with temperature and heating time of the studied samples. The PEURs resisted up to 150 h thermal aging time, after which they became sticky or completely degraded, making color determinations difficult.

One may observe a significant increase in Δ*E* values with temperature as the heating time increases. Apparently the most important color changes occur in the first 50 h of thermal aging. The studied samples darkened when heated. From Figure 5 one may observe two tendencies: (i) at lower temperatures (40 and 70 °C) Δ*E* values slowly increase with aging time, especially up to 150 h, after which they rapidly increase. At temperatures exceeding 100 °C the variations are significant even from the first 50 h. The lowest Δ*E* values were recorded at temperatures up to 70 °C for the samples PEUR 1, 2 and 5 which contain BD chain extender. There seems not to be a major influence from the diisocyanate structure. For the PEURs 3, 4 and 6, containing HD chain extender, the Δ*E* values are situated in the range 20–30. In the time range 150–200 h at 40 °C the Δ*E* values do not exceed the value of 10 for all PEURs. At higher temperature values (100 and 130 °C) and after 50 h of thermal aging the Δ*E* values reach maximum values regardless of structure, suggesting the presence of some significant degradation processes.

In order to estimate the lifetime of the PEURs a simplified form of an Arrhenius equation based on time-temperature relation was used (Equation (2)) [36]. The time (*t*) required for 20% deterioration of a physical property, such as Δ*E*, at various temperatures is used in Equation (2), where *t* is the lifetime, *t*_0_ is the pre-exponential factor, *E* is the activation energy, *T* is the aging temperature and *R* is the gas constant.

*t =t*_0_*e^E/RT^* (2)

Aging times that correspond to an increase in Δ*E* value from 0 to 5, when the color differences became visible, are shown in Table 3, together with kinetic parameters values obtained from the Arrhenius plots.

#### 3.2.2. Thermal Behavior of the Aged PEURs

##### TGA in Non-Isothermal Conditions

In general, during thermal aging, polymers usually undergo chain scission, cross-linking or both. In order to obtain more in-depth information concerning the PEURs structures after thermal aging, thermal analysis was also implied, through correlating the TGA and DSC data. TGA experiments in air for the six PEURs were reported in a previous paper [21]. In general, as expected, depending on the chain extender, in air atmosphere the samples thermally degraded at lower temperature ranges (250–500 °C) and in three stages, compared to the TGA study in nitrogen, described below. The global mass loss values were also considerably higher in air atmosphere, leaving significantly lower residue values.

**Figure 6:**
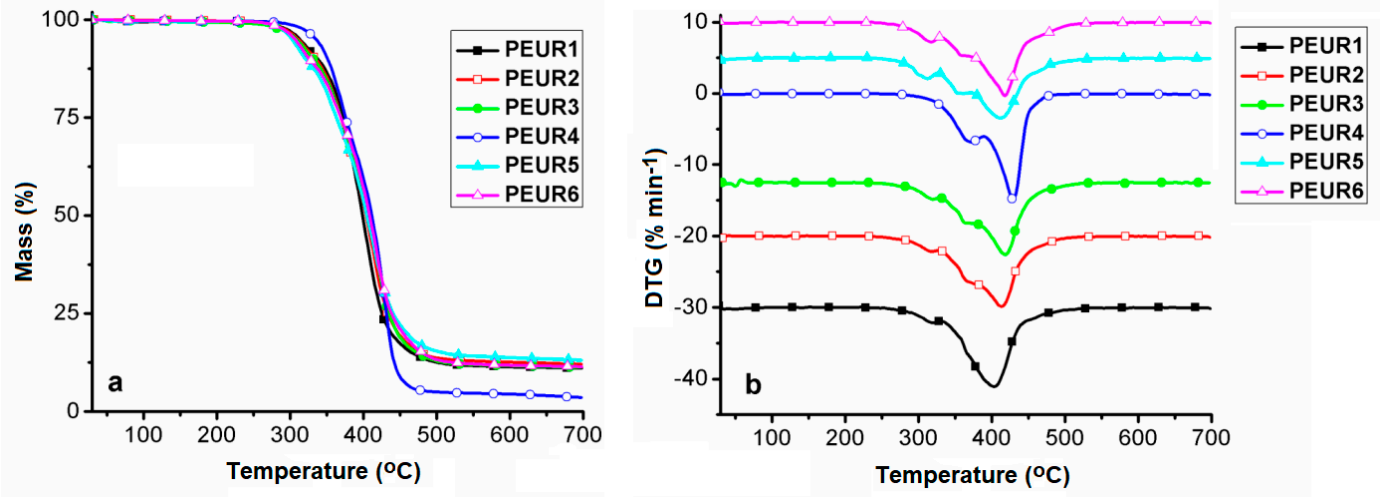
TG (**a**) and DTG (**b**) curves of the studied PEURs.

Figure 6 shows the TGA curves (Figure 6a) and their corresponding first derivatives (DTG) (Figure 6b). Experiments were conducted in inert atmosphere (nitrogen) in order to avoid oxidation and thus secondary undesired reactions during thermal degradation of the aged samples. Data extracted from these curves are given in Table 4. The temperature corresponding to 5% mass loss (*T*_5%_) was used as criterion for evaluating the thermal stability of the samples.

Regardless of their composition, PURs are generally known as being thermally unstable, since the urethane bond’s initial degradation temperature is dependent on the structure. In light of this aspect, the urethane bond may undergo three general thermal decomposition pathways, either individually or simultaneously. In a first instance, the urethane moiety witnesses scission to generate isocyanate and alcohol entities. A second thermal degradation pathway consists of the yielding of olefin and primary amines. The third instance resides in the formation of secondary amine and carbon dioxide evolvement [37,38].

Figure 6 and Table 4 show that the samples respect a four-stage decomposition pattern, except for PEUR4, which degrades in three stages and exhibits the highest thermal stability (*T*_5%_ = 335 °C). Depending on their structures and the processes occurring during thermal aging, the PEURs thermal stability increases in the following order: PEUR5 < PEUR6 < PEUR3 < PEUR2 < PEUR1 < PEUR4. According to the thermal degradation data, in the first stage, samples start decomposing in the temperature range 304–335 °C, not uncommon for PURs, ending in the range 322–390 °C and with mass loss values in the interval 7.97–33.5%, the highest being registered for PEUR4, attributed to urethane bonds cleavage. Cleavage of urethane bonds further initiates depolymerisation with hard segment decomposition in the second stage [39]. This is followed by complex degradation through random scissions of polymer chains together with soft segments in the third and fourth stages [40,41]. For sample PEUR4, the second and third stages coalesce into a single one and with a very high cumulated mass loss (54.21%). This aspect, together with the highest recorded *T*_5%_ value is an indication of more intense cross–linking, compared to the other PEURs. The residual mass (*M*_rez_) is very dependent on the hard segment structure, rich in chains of 6 CH_2_ from HDI and HD which exhibit different behavior during thermal degradation. *M*_rez_ values vary in the range 3.52 (PEUR4)–13% (PEUR5) (Table 4).

**Figure 7:**
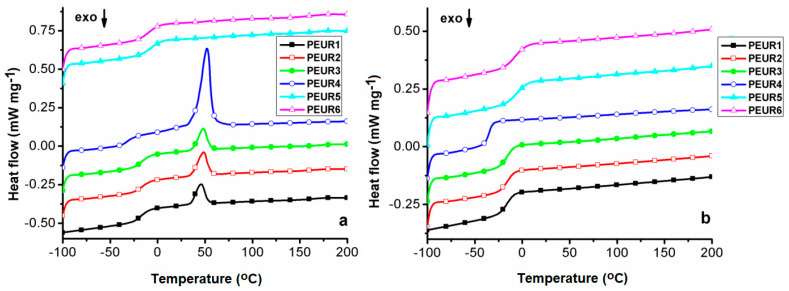
DSC first heating (**a**) and second heating (**b**) curves of the aged PEURs.

Figure 7 shows the DSC first heating (Figure 7a) and second heating curves (Figure 7b) of the studied compounds.

Data extracted from the DSC measurements are given in Table 4. The DSC heating curves present only the glass transition temperature (*T*_g_), associated with the soft segments, from −33 to −5 °C on the first heating, and from −35 to −7 °C on the second heating, due to little influence from hard segment domains and/or cross-linking. The lowest *T*_g_ values were recorded for the sample PEUR4. This signifies that PEUR4′s longer chain components endow the structure not only with enhanced cross-linking capacity, as shown from TGA data, but also with flexibility, due to more free volume between chain segments. As expected, the highest *T*_g_ values correspond to the more rigid aromatic structures of the PEUR5 and PEUR6 structures.

Furthermore, the two PEURs do not exhibit any other transition than the *T*_g_ specific to the soft segment domains. All the other structures show an endothermic peak, associated with a melting process (*T*_m_) [42], in the range 46 °C–52 °C. However, these neither exhibit corresponding crystallization peaks on the cooling curves (not shown), nor melting reproducibility on the second heating curves, this being another aspect related to cross-linking phenomena. The highest *T*_m_ (52 °C) and melting enthalpy (Δ*H*_m_ = 36.84 J g^−1^) values registered for sample PEUR4 demonstrate that its aliphatic chains from hard domains possess the highest capacity to form structural crystalline domains. The other three structures show significantly reduced Δ*H*_m_ values, compared to PEUR4, with almost negligible differences between them (10.67 J g^−1^ for PEUR2, 9.039 J g^−1^ for PEUR3 and 8.141 J g^−1^ for PEUR1). Another aspect which may be due to cross-linking phenomena resides in the fact that none of the PEURs structures exhibited the so called exothermic “cold crystallization” transition on either of the heating curves. This transition is related to the minor structural reordering in soft segments [43].

##### TGA in Isothermal Conditions

**Figure 8:**
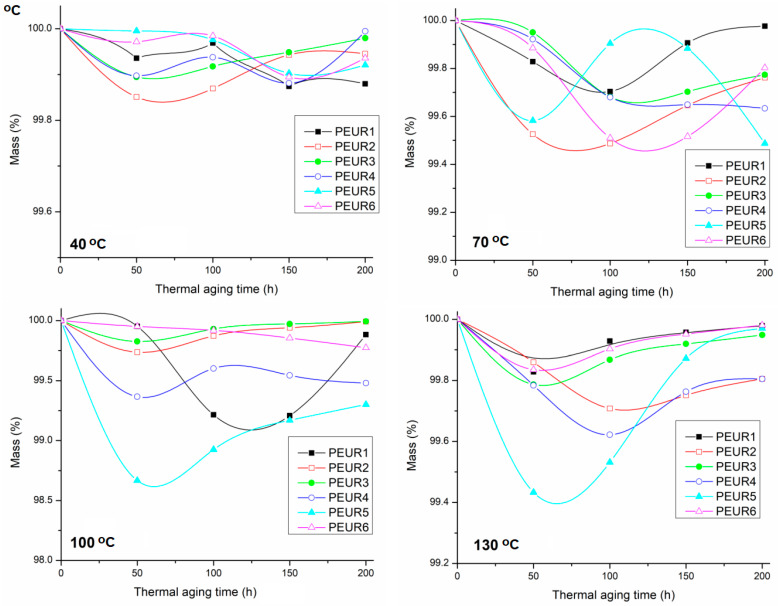
Evolution of mass variation of thermal aged PEURs at 40, 70, 100 and 130 °C.

The percent of mass variation as a function of isothermal aging time is given in Figure 8.

Figure 8 shows that the PEURs are generally thermally stable up to 200 h aging time. Slight mass losses (0.1–0.6%) occur in the range 50–100 h, followed by avery slight increase. This is most probably due to some oxidation phenomena. In this case, no specific pattern was found in mass variation during thermal aging. Additionally, mass loss percentages are generally very low. Furthermore, the DSC data show seemingly increased *T*_g_ values generally compared to non aged PEURs [21]. This means that cross-linking processes compete with chain scissions, owed to complex degradation phenomena.

### 3.3. Structural Modifications during Thermal Aging

**Figure 9:**
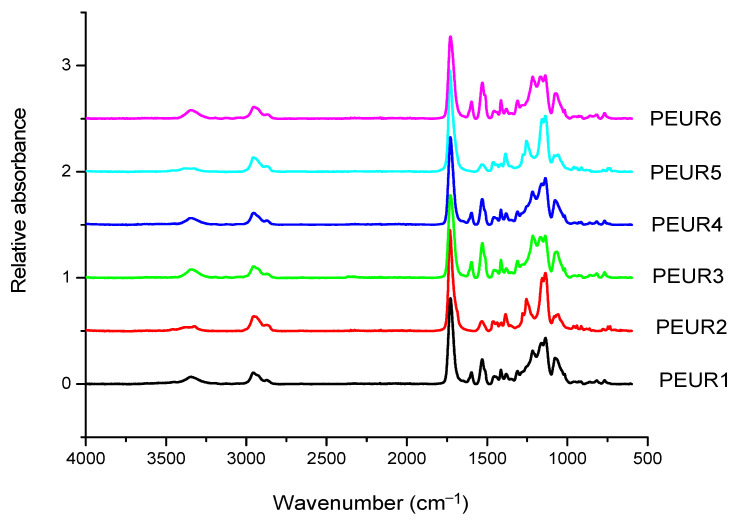
FTIR spectra of PEURs after thermal treatment 200 h at 130 °C.

**Figure 10:**
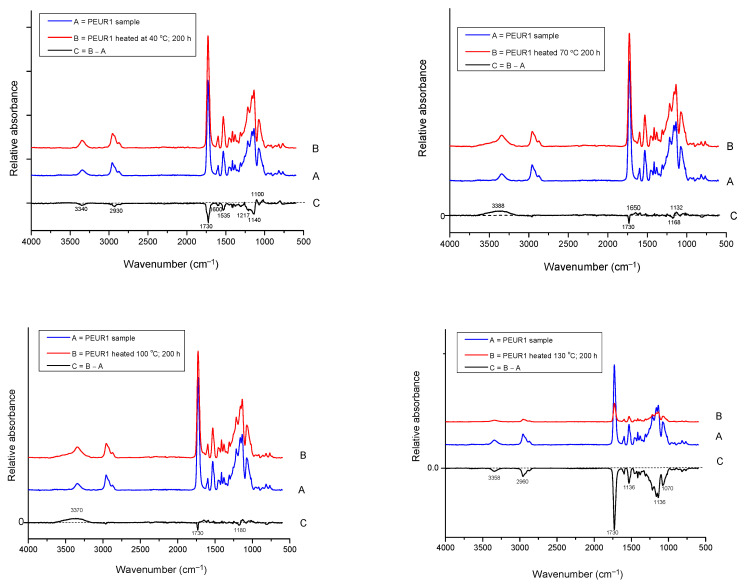
FTIR spectra of PEUR1 before and after thermal aging 200 h at: 40, 70, 100 and 130 °C and the difference spectrum.

The FTIR analysis showed that the changes in structural properties are generated by some structural modifications in the PEURs. As TGA indicated high thermal stability (approx. 300 °C), no major structural changes were observed during the thermal aging of PEURs (Figure 9). In this regard, Figure 10 shows an example of the specific changes in FTIR spectrum of PEUR1 after aging 200 h at the specified temperatures. It can be generally observed from Figure 10 that loss of urethane linkages occurs during heating, accompanied by the enlargement of the band from 3340 cm^−1^, characteristic to the urethane NH moiety, and the slight decrease in the signal from 1730 cm^−1^, specific to the urethane carbonyl group. The urethane group was cleaved during the thermal aging process. The difference FTIR spectra generated mainly medium intensity negative signals (the downward signals) due to the loss of carbonyl groups during thermal aging (1200–1100 cm^−1^). These highly unstable moieties are responsible for both color and mass variations.

### 3.4. Mechanical Properties

The mechanical behavior of PUREs depends on the intermolecular interactions between the hard segments. Physico-mechanical analyses show the extent of supramolecular changes in PURs occurred depending on their structural changes. Hard segments act as multifunctional connecting points that function both as physical and chemical cross-links, while soft segments form an elastomeric matrix determining the elastic properties of PEURs. Three-component PEURs are organized into hard and soft segments consisting of isocyanate and chain extender on the one hand, and PEA chains on the other. The sequence distribution of these segments may also change with the composition and separation of the influence phases, as well as with the formation of the hard and soft phases. In addition, physical cross-linking through the rigid parts of diphenyl methylene also contributes to the maximum stress. In general, the superior mechanical properties of PEURs are the result of microphase separation. The rigid segment acts as a multifunctional cross-linker and is very important for the mechanical properties of the material. In general, the behavior of these materials depends on the size and concentration of rigid segment domains, the strength of the aggregation of rigid segments and the ability of elastic segments to crystallize under deformation. The length of the elastic segments also has a strong influence on the mechanical properties. Short elastic segments generally yield rigid materials with high modulus while long elastic segments give highly elastic materials that can induce crystallization of elastic segments under applied stress. The synthesized compounds, subjected to mechanical deformation in static regime, have both elastomeric and plastic behavior. The mechanical parameters depend on the structure and defects of the analyzed sample. The rigid domains of elastomers are responsible for their “strength”. Their influence is strong because they are deformable and evenly distributed in the sample volume. The plastic deformation of the rigid domains reduces the concentration of internal energy, the result being the increase in the resistance and hardness of the tested material.

Strength, modulus and elongation are important for characterizing polymers depending on their structure. At room temperature, the PEA polymer is in a glassy or partially crystalline state (*T*_g_ = −42 °C). Thus, flexible PEA-based segments induce greater elongations than other PURs. The introduction of chemical cross-linking in the PEUR structure prevents the mutual movement of the macromolecular chains subjected to deformation. Increasing the concentration of polymer bonds leads to an increase in the interpenetration of macromolecules and an overlap of chain fragments with the intensification of intermolecular bonds.

The mechanical properties of cross-linked PEURs were analyzed following the influence of the structure, quantity and length of the rigid segments (diisocyanate and chain extender). PEA at ambient temperature is in the solid state due to the fact that it possesses ester bonds in the structural unit. Its use in the synthesis of urethane copolymers should contribute to increasing the flexibility of chains undergoing mechanical testing. The tensile properties of PEURs block copolymers with various cross-linked hard segment content are shown in Table 5.

The physico-mechanical measurements whose results are presented in Table 5 show that an increase in the ratio of rigid segments in the polyesters leads to a decrease in tensile strength. Thus, an increase in the ratio of diisocyanate groups from 2 to 3 and chain extender groups from 1 to 2 leads to a decrease in breaking strength of about two times. The same can be seen in the case of the elongation at break where there is a decrease from 500% to 200%. This can be explained by the formation of hydrogen bonds due to the structure of the hard domains.

Therefore, continuing to increase the deformation for PEA, the forces that prevent the deformation by rearranging the macromolecules also increase. In tetramethylene monomer units, only hydrogen atoms are attached to the molecular chain.

The high molecular symmetry and mobility of the chain segments make them partially crystalline at room temperature. Because crystalline regions play a similar role to cross-linking in improving mechanical properties, the properties of crystallizable materials are superior to non-crystallizable ones. Varying the molecular masses of the small diol segment affects the physical and mechanical properties of PURs. In PUR segments, the mechanical properties are generally credited as a result of the pseudo reticular effects of hard segment aggregation. The domains of the hard segments develop different degrees of ordering or semicrystalline structures, which have been increased in the case of PURs by adding the chemical cross-linking effect.

The presence of amorphous materials can also be established within the domain of hard segments. Pseudo-cross-linking failure can begin in this region. From an engineering point of view, the final mechanical parameters of the synthesized PEURs (strength, elongation and breaking modulus) are of particular interest.

**Figure 11:**
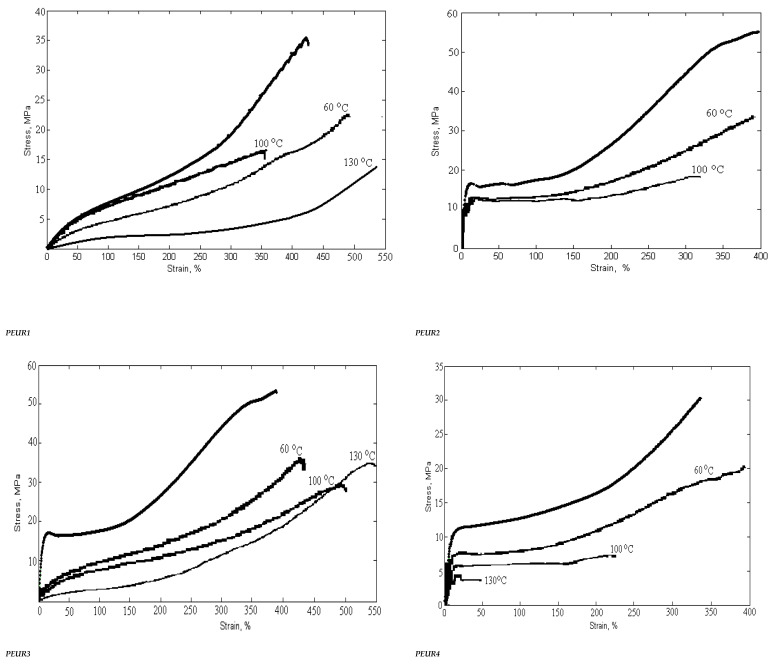

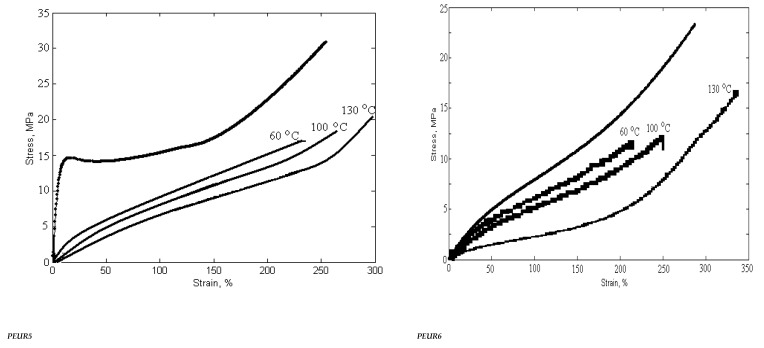
Variation of physico-mechanical properties with temperature and heating time.

These are indicators that recommend the use of PEURs at static or dynamic loads. Long elastic segments and the appropriate hard segment content are required for PEURs with high final shape recovery. This confirms the existence of interactions between soft and hard segments in the molecular chain. The interaction becomes strong with the growth of hard segments. These indicate that there is a strong interaction or existence of physical crosslinks that lead to shape memory. Stabilization of PEURs by hydrogen bonds and induced dipole-dipole interactions of hard segments appear to be responsible for shape memory. It can be concluded that 96–98% of the maximum shape recovery can be obtained at a concentration of 15–35 wt% hard segments when the PEUR copolymer has enough tightly interacted hard segments capable of restoring it to its original shape. Shape memory PURs can be used to manufacture products with both deformation control and damping capacity (Figure 11).

## 4. Conclusions

Six PEURs were subjected to thermal aging at temperatures of 40 °C, 70 °C and 130 °C for 200 h. The FTIR spectra have indicated urethane bond scission during the thermal treatment. The destruction of the urethane bond advanced with temperature and exposure time increase. The lifetime prediction of the studied samples was conducted following the color changes during the thermal aging. The lifetime calculation was made with an Arrhenius type relation. The structural changes were evidenced by FTIR spectroscopy. The best results were obtained for PEURs synthesized from 4,4′-diphenyl methane diisocyanate and 1,4-butanediol. The evolution of the mechanical properties coincides perfectly with the structural variations, chemical changes deduced from the FTIR and thermal characterization. The degradation of the PEURs was essentially manifested by modification in the intensity of some urethane specific signals. From the mechanical analysis, it was concluded that maximum shape recovery, up to 96–98% may be obtained at a concentration of 15–35 wt% hard segments, when the PEURs develop sufficient physical interactions capable of restoring their original shapes after mechanical deformation. Sample mass variation of PEURs during isothermal aging was analysed. All characterization results were in good agreement and showed a variation in thermal, mechanical, optical properties and mass. This led to a competition between scission and cross-linking reactions during thermal aging.

## Figures and Tables

**Table 1:** The composition of the poly(ester urethane) elastomers (PEURs.)

Sample	Ratio Polyester/Diisocyanate/ Chain Extenders + Glycerin	Chain Extenders	Diisocyanate	Hardness (Shore A)
PEUR1	1:2:1	BD	MDI	46
PEUR2	1:2:1	BD	HDI	93
PEUR3	1:2:1	HD	MDI	48
PEUR4	1:2:1	HD	HDI	93
PEUR5	1:3:2	BD	MDI	62
PEUR6	1:3:2	HD	MDI	53

**Table 2:** Color parameters variation during thermal aging.

Heat Treated (°C)/Time (h)	PEUR1			PEUR2			PEUR3			PEUR4			PEUR5			PEUR6		
	ΔL^*^	Δa^*^	Δb^*^	ΔL^*^	Δa^*^	Δb^*^	ΔL^*^	Δa^*^	Δb^*^	ΔL^*^	Δa^*^	Δb^*^	ΔL^*^	Δa^*^	Δb^*^	ΔL^*^	Δa^*^	Δb^*^
**40 °C**																		
0	0	0	0	0	0	0	0	0	0	0	0	0	0	0	0	0	0	0
50	−1.32	0.53	1.59	2.51	−0.80	−2.27	−2.66	−1.64	−3.01	4.77	0.42	7.12	−2.76	2.68	−1.38	−1.04	−2.05	7.33
100	2.51	−0.33	0.64	−3.25	−0.47	−6.28	−3.60	−5.80	−5.03	3.01	−1.90	6.39	−5.09	0.29	0.48	−2.29	−5.64	2.49
150	0.08	−0.15	2.29	−3.89	2.13	−5.86	−3.86	−2.57	−5.41	−1.38	0.58	5.12	−6.17	−1.30	0.12	−6.06	−3.88	4.28
200	−3.68	−1.88	1.68	25.70	−1.58	−0.19	−2.37	−1.71	−5.62	38.06	−3.97	4.25	−1.32	0.79	1.18	−2.83	−1.39	5.38
**70 °C**																		
0	0	0	0	0	0	0	0	0	0	0	0	0	0	0	0	0	0	0
50	1.88	−1.09	3.89	2.07	1.16	1.73	3.46	−3.41	6.22	−9.13	10.24	2.89	4.34	−1.33	3.98	4.12	−5.66	6.28
100	5.03	−2.71	4.36	4.09	2.35	2.02	7.17	−5.49	13.34	4.71	2.72	3.65	7.24	−3.16	6.58	6.56	−3.99	10.3
150	−5.60	−4.68	5.73	−3.54	0.16	2.45	−3.56	−1.81	19.91	−2.73	−0.60	3.67	9.42	−4.49	8.09	−3.12	0.21	6.54
200	19.75	−3.73	14.17	33.83	3.34	5.26	20.61	−2.07	19.21	36.62	−8.40	7.64	22.56	−2.63	14.37	20.02	−5.57	20.60
**100 °C**																		
0	0	0	0	0	0	0	0	0	0	0	0	0	0	0	0	0	0	0
50	−31.83	14.83	18.57	−43.91	4.13	−14.55	42.94	10.37	23.71	−44.11	5.36	−16.74	−24.47	1.68	19.61	−24.95	8.35	17.64
100	−33.65	18.73	18.28	−43.20	3.31	−13.40	42.89	9.74	31.07	−45.21	4.5	−18.38	−25.74	6.59	20.49	−28.80	8.94	21.30
150	−39.73	18.15	17.27	−43.78	2.51	−12.23	36.42	6.71	28.17	−43.13	2.01	−16.72	−29.86	6.24	20.47	−34.49	9.36	17.92
200	−41.64	17.44	18.3	−42.82	0.26	−4.19	33.87	11.42	30.37	−42.58	1.94	−10.51	−34.04	9.39	21.13	−36.88	16.11	22.92
**130 °C**																		
0	0	0	0	0	0	0	0	0	0	0	0	0	0	0	0	0	0	0
50	−27.66	24.71	16.17	1.18	−3.96	13.92	−31.63	19.59	25.35	−3.01	−3.37	20.74	−18.06	24.87	10.64	−17.70	24.69	25.05
100	−36.07	29.30	10.30	−13.56	−1.83	18.61	−38.16	26.34	18.74	−5.36	1.97	33.91	−24.47	28.78	5.78	−24.37	27.79	19.13
150	−38.34	32.36	4.73	−18.80	4.50	16.16	−41.53	28.78	16.01	−10.3	6.30	19.04	−30.25	30.98	−3.17	−29.25	25.89	10.12
200	-	-	-	-	-	-	-	-	-	-	-	-	-	-	-	-	-	-

**Table 3:** Composition, aging times that correspond to the increase in Δ*E* value from 0 to 5 and kinetic parameters obtained from Arrhenius plot for PEURs.

Sample	Aging Time (h)				*E* (kJ mol^−1^)	ln*t*_0_	Correlation Coefficient	Lifetime at 25 °C (h)
	40 °C	70 °C	100 °C	130 °C				
PEUR1	200	63	6	-	67	−19.514	0.967	1568
PEUR2	100	71	17	4	38	−9.580	0.861	306
PEUR3	58	32	5.5	4.8	29	−7.110	0.954	116
PEUR4	29	12	9.5	5.2	19	−3.987	0.949	39
PEUR5	33	27	7.7	6.6	19	−3.955	0.837	127
PEUR6	30	26	9.9	5.1	81	−26.063	0.999	-

**Table 4:** Data extracted from TG, DTG and DSC curves.

Sample				TGA					DSC		
	Stage	*T*_5%_ (°C)	*T*_onset_ (°C)	*T*_max_ (°C)	*T*_f_ (°C)	*M*_m_ (%)	*M*_rez_ (%)	Scan	*T*_g_ (°C)	*T*_m_ (°C)	Δ*H*_m_ (J g^−1^)
PEUR1	I	319	–	324	340	8.02		1st heating	−17	46	8.141
	II	–	349	367	390	17.77					
	III	–	390	404	459	50.48	10.95	2nd heating	−16	–	–
	IV	–	459	482	525	12.19					
PEUR2	I	315	–	322	335	7.97		1st heating	−15	49	10.67
	II	–	344	367	387	27.39					
	III	–	387	422	455	41.17	11.96	2nd heating	−15	–	–
	IV	–	455	480	520	11.46					
PEUR3	I	310	–	320	332	8.58		1st heating	−16	48	9.039
	II	–	340	363	381	21.74					
	III	–	381	421	450	48.36	11.23	2nd heating	−16	–	–
	IV	–	450	478	510	9.31					
PEUR4	I	335	–	372	390	33.5	3.52	1st heating	−33	52	36.84
	II	–	390	429	450	54.21		2nd heating	−35	–	–
	III	–	450	460	500	8.39					
PEUR5	I	304	–	313	322	10.76		1st heating	−5	–	–
	II	–	328	355	374	18.03					
	III	–	374	412	442	45.49	13	2nd heating	−7	–	–
	IV	–	442	465	490	12.24					
PEUR6	I	307	–	318	330	10.29		1st heating	−9	–	–
	II	–	335	358	378	18.46					
	III	–	378	418	444	45.12	11.37	2nd heating	−7	–	–
	IV	–	444	468	503	14.26					

*T*_5%_*—*temperature corresponding to 5% mass loss; *T*_onset_*—*onset thermal degradation temperature; *T*_max_*—*temperature that corresponds to the maximum rate of decomposition for each stage evaluated from the peaks of the DTG curves; *T*_f_*—*final thermal degradation temperature; *M*_m_*—*mass loss rate corresponding to each thermal degradation stage; *M*_rez_*—*percentage of residue remained at the end of the thermal degradation process (700 °C); *T*_g_*—*glass transition temperature; *T*_m_*—*melting temperature; Δ*H*_m_*—*melting enthalpy.

**Table 5:** Variation of physico-mechanical properties with temperature and aging time.

Sample	Heating Temperature, °C	Initial Modulus of Elasticity, MPa	Elongation at the Limit of Elasticity, %	Resistance at the Limit of Elasticity, MPa	Strain at Breaking, %	Resistance at Breaking, MPa
PEUR1	-	0.090	12.248	2.020	420.744	35.240
	40	0.031	20.150	0.523	549.873	16.304
	70	0.076	24.970	1.873	487.376	22.626
	100	0.111	17.768	2.276	357.216	16.645
	130	0.215	14.938	0.319	533.058	13.882
PEUR2	-	4.277	0.574	10.106	394.247	55.702
	40	0.738	4.525	2.951	398.075	20.824
	70	1.592	7.717	11.870	386.895	33.769
	100	1.397	8.288	12.117	303.413	18.402
	130	-	-	-	-	-
PEUR3	0	1.366	2.073	6.552	383.928	54.320
	40	0.059	15.137	0.640	547.353	23.416
	70	0.700	4.145	3.353	424.753	37.060
	100	0.348	6.922	2.473	493.676	31.176
	130	0.062	14.530	1.022	497.580	30.835
PEUR4	-	1.137	1.410	2.923	331.178	31.279
	40	0.354	5.202	4.301	398.075	19.599
	70	0.474	10.289	7.340	387.812	21.310
	100	0.185	11.676	5.562	221.237	7.819
	130	0.162	12.219	3.493	45.452	3.830
PEUR5	-	2.314	3.309	9.557	254.707	31.587
	40	0.059	10.703	1.631	398.991	13.999
	70	0.197	5.773	1.307	233.814	17.480
	100	0.119	11.092	1.121	264.484	18.892
	130	0.056	14.673	0.810	298.556	20.912
PEUR6	-	0.363	3.005	0.985	337.027	16.777
	40	0.030	21.436	0.914	634.810	13.466
	70	0.108	12.989	1.482	210.240	11.483
	100	0.057	13.761	0.933	248.407	12.201
	130	0.042	15.574	0.715	336.597	16.735

## Data Availability

Not applicable.
